# Comparison of MRI and CT in the Evaluation of Unilateral Maxillary Sinus Opacification

**DOI:** 10.1155/2021/5313196

**Published:** 2021-07-09

**Authors:** Elise Chua, Annakan V. Navaratnam, Dominic St Leger, Vincent Lam, Samit Unadkat, Alexander Weller

**Affiliations:** ^1^Departments of Radiology, Northwick Park and Central Middlesex Hospitals, London North West University Healthcare NHS Trust, Watford Road, Harrow HA1 3UJ, UK; ^2^Departments of ENT Surgery, Northwick Park and Central Middlesex Hospitals, London North West University Healthcare NHS Trust, Watford Road, Harrow HA1 3UJ, UK; ^3^Department of Radiology, Manchester Royal Infirmary, Oxford Road, Manchester M13 9WL, UK; ^4^Royal National Throat, Nose and Ear Hospital, University College London Hospitals NHS Foundation Trust, 47–49 Huntley Street, London WC1E 6DG, UK; ^5^Department of Radiology, St George's Hospital, St George's University Hospitals NHS Foundation Trust, Blackshaw Road, Tooting, London SW17 0QT, UK

## Abstract

**Objectives:**

To evaluate the diagnostic performance of MRI compared with CT in differentiating neoplastic from infectious/inflammatory causes of complete unilateral maxillary sinus opacification (UMSO). Although MRI is increasingly used, no studies validate its utility compared to CT or nasal endoscopy in this context.

**Methods:**

A retrospective analysis of 49 patients presenting with complete UMSO to a tertiary referral centre was performed, investigated with both CT and MRI. Two head and neck radiologists independently reviewed each imaging modality and recorded both a final diagnosis and Likert-scale diagnostic certainty score. A consensus radiological diagnosis was determined, stratified into potentially neoplastic or infectious/inflammatory aetiology, and compared with nasal endoscopy and final diagnosis. Diagnostic performance and interoperator agreement for predicting neoplasia were calculated.

**Results:**

Both CT and MRI demonstrated high sensitivity and negative predictive value for neoplasm, although MRI was more specific (79%; 95% CI: 60–92%) than CT (14%; 95% CI: 4–32%), with a higher positive predictive value. MRI was more accurate (88%; 95% CI: 75–95%) than CT (49%; 95% CI: 34–64%) in diagnosing neoplasia. MRI had significantly higher diagnostic certainty Likert scores than CT (*p* < 0.0001 for both observers). Interobserver agreement was fair for CT (kappa coefficient = 0.327) and excellent for MRI (kappa coefficient = 0.918).

**Conclusions:**

MRI is more specific than CT in characterising UMSO, with greater diagnostic certainty and reproducibility. The additive diagnostic value of MRI complements CT, potentially reducing diagnostic delays in some cases and the need for diagnostic endoscopic sinus surgery in others. We recommend MRI incorporation into the diagnostic pathway for patients with UMSO.

## 1. Introduction

**Figure 1:**
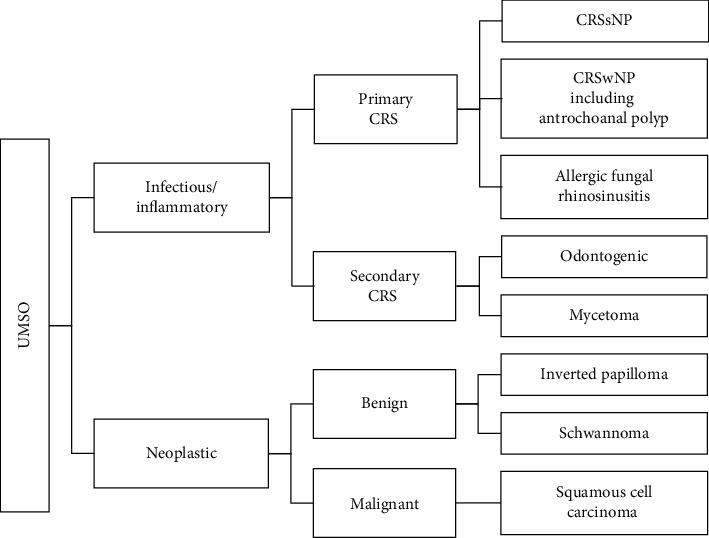
Causes of unilateral maxillary sinus opacification (UMSO). CRS: chronic rhinosinusitis, CRSsNP: CRS without nasal polyps, and CRSwNP: CRS with nasal polyps.

Sinonasal disease requiring clinical and imaging evaluation is common, affecting 5–12% of the population. The majority are due to infection or inflammation, most commonly chronic rhinosinusitis (primary or secondary). Neoplastic causes are rare, but localised disease such as unilateral maxillary sinus opacification (UMSO) is more likely to have neoplastic aetiology than diffuse bilateral disease [1, 2]. Inverted papilloma (IP) and squamous cell carcinoma are the most frequent benign and malignant lesions, respectively (Figure 1).

This is recognised in the 2020 European position paper on rhinosinusitis and nasal polyps (EPOS), which states “*isolated maxillary sinus opacification is a marker of neoplasia in 18% and malignancy in 7–10%*”*...*“*so clinicians should be wary of conservative management and have a low threshold for early surgical intervention*” [3].

For localised obstruction, the primary concern is to differentiate neoplasia from inflammation. However, as symptoms overlap and tumours often have superimposed inflammation, differentiation is challenging clinically [4]. In addition, due to the intricate anatomy, early- and late-stage sinonasal malignancy have vastly different prognoses, leading to considerable medicolegal costs when missed [5]. High sensitivity for neoplasia is therefore crucial, although this is at the expense of low specificity with the current standard of care of low dose CT and flexible nasal endoscopy (FNE) [6, 7].

CT forms the mainstay for sinonasal imaging, providing a low-cost technique for assessing drainage pathways, pattern of obstruction, and bony structures. However, other than inference from Hounsfield-unit densities, intralesional soft tissue characterisation is limited [6, 7]. Aggressive features such as bony erosion or fat infiltration are seen with both benign and malignant aetiologies [8] and may be absent at early stages. Thus, even when heralding malignancy, the diagnosis may be at an advanced stage [9, 10].

FNE is performed for the majority of patients referred to otolaryngology clinics with rhinological symptoms. Although able to identify meatal obstruction by nasal polyps or oedematous mucosa, its role in UMSO is limited due to restricted views of middle meatus, little to no visualisation of the maxillary ostium, sinus contents, and consequent failure to demonstrate abnormalities deep to middle meatus [3, 11, 12].

Due to the relatively high baseline risk of malignancy, a large proportion of patients with UMSO on CT require further investigation with endoscopic sinus surgery (ESS) for histological confirmation regardless of FNE findings [13]. This results in multiple surgical procedures (diagnostic and therapeutic), prolonging time to diagnosis as well as treatment.

MRI is used extensively in the head and neck and potentially provides superb soft tissue characterisation for sinonasal obstruction. It can delineate tumour from secretions and define invasion of adjacent structures, including crucially at the skull base and orbit [14]. If MRI was confirmed to have a greater diagnostic accuracy than CT in differentiating neoplastic from infectious/inflammatory causes of UMSO, it could avoid missed diagnoses of malignancy (improving patient triage to urgent ESS for histological confirmation) or increase the number of confident benign diagnoses, avoiding unnecessary ESS.

Whilst MRI is increasingly used in clinical practice prior to ESS, limited data exists comparing its diagnostic performance with CT or FNE. The aim of this study was to evaluate the diagnostic accuracy of MRI compared with CT (and FNE) in differentiating potentially neoplastic from infectious/inflammatory causes of UMSO (and by extrapolation sinonasal disease in general), to determine which patients warrant subsequent ESS. A secondary aim was to assess interobserver agreement for image interpretation between radiologists, to determine the level of diagnostic reproducibility.

## 2. Materials and Methods

Patients referred to a tertiary rhinology and head and neck oncology centre prior to January 2017, with complete UMSO detected on CT that proceeded to MRI ± ESS (under our standard institutional diagnostic pathway), were included. As this study comprised a retrospective analysis of data acquired for standard clinical care, ethics committee review and informed consent were waived. Data were collected on clinical, operative, and histological reports. FNE findings were categorised as potentially neoplastic (f-N) or infectious/inflammatory (f-I). For this study, “f-N” was recorded if reported in the operative notes as possibly neoplastic *or* equivocal, as both required further evaluation. To avoid missing potentially malignant sinus obstruction, “f-I” was recorded *only* if reported in the operative notes as such.

The final diagnosis was determined from findings at ESS and histology or diagnosis of CRSsNP based on clinical features and nasal swab (as per EPOS guidelines) and stratified into infective/inflammatory (c-I) or neoplastic (c-N).

### 2.1. CT and MRI Protocol

CT scans were acquired using Philips Ingenuity 128 (Philips Medical Systems, Eindhoven, The Netherlands) or Toshiba Aquilion 32 (Canon Medical Systems, Sussex, United Kingdom) systems with tube voltage 120 kV, current 30 mA, and patient positioned with an occlusal plane parallel to the gantry, with 500 mm field of view, from maxillary alveolus to top of the frontal sinuses. Images were reconstructed using high frequency (bone) and low frequency (soft tissue) convolution kernels to 0.9 mm slices (0.45 mm interval), with each 150–200 mm diameter reconstructed image presented in a 512 × 512 matrix.

MRI studies were performed on Siemens Avanto (Siemens AG, Erlangen, Germany) or Philips Achieva (Philips Medical Systems, Eindhoven, The Netherlands) 1.5T platforms with 6- or 8-channel phased array head and neck coils, according to departmental protocol. This included axial 3D isotropic T1-weighted (T1W) (repetition time (TR)/echo-time (TE) = 500/8.2 ms and slice thickness = 0.9 mm), axial 3D isotropic short-tau inversion recovery (STIR) SPACE (TR/TE = 2150/199 ms, inversion time (TI) = 150 ms, and slice thickness = 1.1 mm), axial and coronal 2D T2-weighted (T2W) turbo spin-echo (TSE) (TR/TE = 5730/83 ms, slice thickness = 3 mm, and 0.6 mm interspace), axial 2D echo-planar diffusion-weighted imaging (TR/TE/TI = 7080/81/130 ms, slice thickness = 4 mm, 1.6 mm interspace, b-factors of 50 and 800 s/mm^2^, and calculated apparent diffusion coefficient), and axial 3D isotropic contrast-enhanced T1W gradient echo fat-saturated images (TR/TE = 6/2.81 ms and slice thickness = 0.8 mm, with spectral attenuated inversion recovery (SPAIR) fat saturation) and acquired 100 seconds following gadolinium administration (Dotarem, Gerbet Pharma, Villepinte, France). Where 3D sequences were not possible or degraded by motion, our technicians reimaged with 2D T1W TSE, STIR T2W, and T1W contrast-enhanced fat-suppressed sequences, due to higher temporal resolution afforded by acquiring each slice individually.

### 2.2. Imaging Analysis

Anonymised images were independently analysed on a proprietary PACS system (Sectra PACS, Linkoping, Sweden), by two consultant head and neck radiologists, Observer1 and Observer2, each with a four-year subspecialty experience. CT and MRI images were evaluated independently of each other and more than 72 hours apart, so diagnoses made were based solely on each study in isolation.

Each observer recorded the overall radiological diagnosis as “infective/inflammatory,” “equivocal,” or “likely neoplastic.” “I” diagnosis only resulted when confident of benign aetiology, with no concern for possible neoplastic or “equivocal” differential. So as not to miss malignancy, “equivocal” or “likely neoplastic” diagnoses were classified as potentially neoplastic “N,” as both required further characterisation.

Imaging features were also assessed over three domains: soft tissue characterisation, localisation, and ability to evaluate secondary structures (Table 1). A 10-point Likert score for diagnostic certainty was calculated from this, where features scored “1” if defined (definitely present or absent) and “0” if indeterminate.

A consensus diagnosis was determined, where “r-I” resulted only if recorded as “I” by *both* observers. If one or both observers recorded as “equivocal” or “likely neoplastic,” the consensus was graded as such (r-N), as practically further evaluation with biopsy would be required.

### 2.3. Statistical Analysis

Statistical analysis was performed using GraphPad Prism version 6 (GraphPad Software Inc., CA, USA) and Microsoft Excel, where all appropriate tests were two sided, using a significance level of *α* = 5%.

Sensitivity, specificity, positive (PPV), negative predictive values (NPV), and diagnostic accuracy of each modality for predicting final clinical diagnosis were calculated. The performance of CT and MRI for differentiating potentially neoplastic UMSO was represented graphically using receiver-operating-characteristic (ROC) analysis plots and the area under the curve (AUC) was calculated.

To assess the absolute difference between CT and MRI, Chi-squared analysis was performed for the diagnoses reached by each observer and consensus. Diagnostic certainty Likert scores were compared between modalities using Mann–Whitney U test.

Interobserver agreement was calculated using Cohen's Kappa coefficient (*k*) and was interpreted as proposed by Landis and Koch [15]: kappa value of 0 indicates no agreement, 0 to 0.2 slight agreement, 0.21 to 0.4 fair agreement, 0.41 to 0.6 moderate agreement, 0.61 to 0.80 substantial agreement, and 0.81 to 1.0 excellent agreement.

## 3. Results

Patient demographics and final diagnoses are recorded in Table 2. Forty-nine patients with complete UMSO on CT who also underwent MRI were identified, comprising 33 male and 16 female, with a mean age of 51 years (range 16 to 83 years). Twenty-nine (59%) patients had a final diagnosis of infective/inflammatory aetiology (c-I), of which twenty-one had a diagnosis of CRS: CRS without nasal polyps (CRSsNP) (*n* = 6) and CRS with nasal polyps (CRSwNP) (*n* = 12), noninvasive fungal sinusitis (*n* = 2), and acute invasive fungal sinusitis (*n* = 1). Odontogenic sinusitis was observed in two cases of both CRSsNP and CRSwNP and antrochoanal polyps in four cases of CRSwNP.

Twenty (41%) patients had neoplastic diagnoses (c-N), most frequently IP (*n* = 9), and malignancy (*n* = 8). Forty-four patients underwent surgery. The remaining five who underwent medical treatment alone had a diagnosis of CRSsNP.

### 3.1. Diagnostic Accuracy

The diagnostic accuracies of MR and CT in identifying potentially neoplastic aetiology, for Observer1, Observer2, and consensus, are recorded in Tables 3 and 4. Most importantly, all 20 cases with a final diagnosis of neoplasia were correctly classified on both CT and MRI, by both observers and at consensus. Using this threshold, both modalities had 100% sensitivity and 100% NPV for identifying neoplasia.

However, for CT, this was at the expense of low specificity (14%, 95%CI: 4–32%) and PPV (44%, 95%CI: 41–48%) at consensus, compared with specificity at MRI of 79% (95%CI: 60–92%) and PPV of 77% (95%CI: 62–87%)). This higher specificity and PPV of MRI are corroborated by the consistently higher number of cases correctly recorded at MRI as “infective/inflammatory,” plus lower number of “potentially neoplastic” diagnoses, as a result of the increased diagnostic confidence afforded at MRI. Confirming this, chi-squared analyses showed a significantly higher number of “I” diagnoses and lower number of “N” diagnoses based on MRI compared with CT, for both observers and at consensus (Observer1 *p*=0.006, Observer2 *p*=0.0002, and consensus *p* < 0.00002).

A similar comparison between FNE and imaging showed no difference between CT and FNE (*p*=0.34), whereas MRI correctly recorded a significantly higher number of infective/inflammatory and lower number of potentially neoplastic diagnoses, confirmed by a Chi-square statistic *p* value of <0.0005. Corroborating these findings, FNE had similar sensitivity for identifying potential neoplasia to both imaging modalities, with comparable specificity, PPV, and diagnostic accuracy to CT, but MRI again demonstrated higher specificity, PPV, and diagnostic accuracy (Table 4).

**Figure 2:**
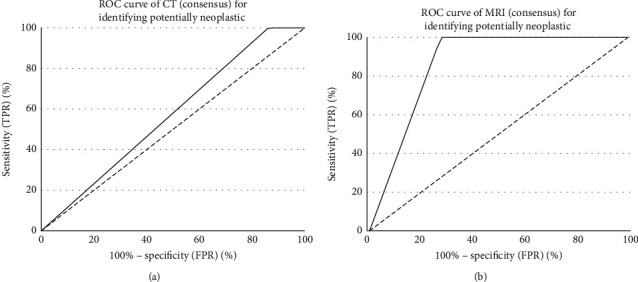
Receiver-operating-characteristic (ROC) curves for differentiating potentially neoplastic from infective/inflammatory causes of unilateral maxillary sinus opacification, for consensus radiological diagnoses: (a) on CT and (b) on MRI. X-axis: false positive rate (FPR), Y-axis: true positive rate (TPR).

From Table 3, at CT, only 4 cases were confidently identified as infective/inflammatory at consensus, meaning that 25 required further characterisation. In contrast, a confident and correct diagnosis of infective/inflammatory aetiology was made at MRI for 23 cases by Observer1, 24 cases by Observer2, and 23 cases at consensus. These consensus results are represented graphically in Figure 2, where the ROC analysis AUC is greater for MRI (0.890) than CT (0.569).

**Figure 3:**
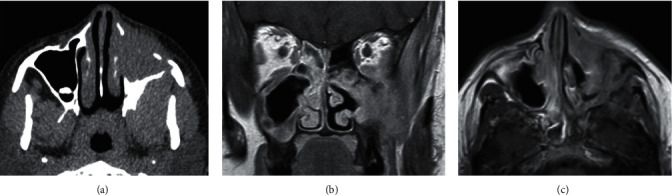
IgG4 disease. A 31-year-old female presented with epistaxis and medial-gaze diplopia. (a) Axial unenhanced CT shows soft tissue opacification of the left maxillary antrum with destruction of the antral walls and lateral pterygoid plate. (b) Coronal and (c) axial contrast-enhanced T1W demonstrates extension of the abnormally enhancing soft tissue (with restricted diffusion, not shown) into the orbit, pterygopalatine fossa, and involvement of the left infraorbital nerve.

Six cases were incorrectly assigned by MRI at consensus to “potentially neoplastic”: two CRSwNP; one invasive fungal sinusitis; one IgG4-related disease; one mucormycosis; and one arteriovenous malformation. Apart from the two misdiagnosed CRSwNP, the remaining cases are transspatial aetiologies with aggressive imaging features (Figure 3).

Where CRS was the final diagnosis, both observers correctly recorded CRSwNP (*n* = 12) or CRSsNP (*n* = 6) in a large majority (16/18) on MRI. Both observers correctly identified IP in 6/9 cases on MRI, compared with only 1/9 and 2/9 at CT for Observer1 and Observer2, respectively.

Interobserver agreement for differentiating neoplastic (N) from inflammatory (I) aetiology was fair for CT (*κ* = 0.327) and excellent for MRI (*κ* = 0.918).

### 3.2. Diagnostic Certainty

The mean diagnostic certainty Likert-score was 6.6/10 for CT and 8.6/10 for MRI for Observer1 and 5.2/10 for CT and 9.4/10 for MRI for Observer2. MRI had significantly higher scores than CT for diagnostic certainty criteria for both observers (*p* value <0.00001).

## 4. Discussion

Our results demonstrate the superior diagnostic performance of MRI compared to CT and FNE in characterising UMSO, with excellent interobserver agreement for differentiating neoplasia from inflammation. These results arise from improved soft tissue characterisation, localisation (by differentiating soft tissue from obstructed sinus contents), and ability to evaluate secondary structures, as reflected by significantly higher diagnostic certainty Likert scores for MRI. To achieve this, the MRI protocol should include anatomic (T1W, T2W, and contrast-enhanced sequences) and diffusion-weighted imaging (DWI), with dynamic contrast for suspected vascular pathology. 3D GRE isotropic fat-suppressed postcontrast sequences are particularly helpful due to excellent spatial resolution and opportunity for multiplanar reconstruction.

Although MRI, CT, and FNE had similar sensitivity for detecting malignancy (100%, 100%, and 85%, resp.), CT and FNE both had low specificity and high false positive rates (FPR). The smaller number of “equivocal” results at MRI yielded both (a) significantly improved specificity and PPV and (b) consistent interpretation between observers. MRI demonstrates improved specificity compared to CT (79% (95%CI: 62–87%) versus 14% (95%CI: 4–32%)) and increased diagnostic accuracy (88% (95%CI: 75–95%) versus 49% (95%CI: 34–64%)). The proportion of neoplastic and inflammatory disease in our patient cohort is comparable to that in the literature and reflects a relatively high incidence of atypical lesions in unilateral disease [2, 16]. These results demonstrate the utility of MRI in directing management, with diagnoses that are reproducible between different radiologists and therefore between institutions.

**Figure 4:**
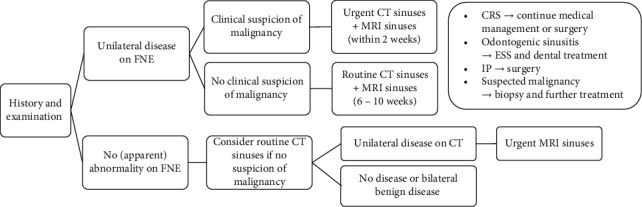
Example diagnostic pathway for unilateral maxillary sinus opacification. Flexible nasal endoscopy (FNE), chronic rhinosinusitis (CRS), endoscopic sinus surgery (ESS), and inverted papilloma (IP).

MRI correctly identified all neoplasms as equivocal or concerning for malignancy, requiring further ESS evaluation. As for CT and FNE, this confirms that MRI is an extremely safe test. Additionally, the lower FPR means that MRI is significantly able to triage patients with inflammatory aetiology to medical therapy better than CT or FNE, potentially sparing the need for general anaesthesia and ESS. Where the cause of UMSO is uncertain at CT or FNE, MRI comprises a low-cost noninvasive investigation with superior diagnostic accuracy in differentiating both (a) malignancy from benign obstruction and (b) benign neoplasms requiring urgent surgery (such as IP) from those in which medical treatment can be trialled (such as CRS). Thus, MRI has a valuable role when used in conjunction with CT for patient selection for surgical management and is routinely included in our institutional pathway in this context (Figure 4).

**Figure 5:**
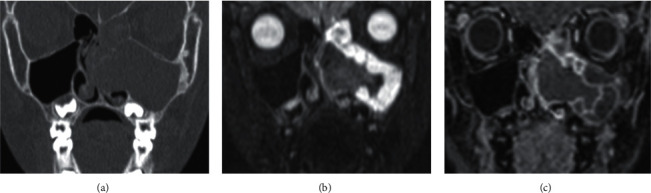
Fungal sinusitis in 7-year-old male with symptoms of nasal obstruction. (a) Coronal unenhanced CT demonstrates left ostiomeatal pattern opacification with maxillary mural osteitis plus expansion of maxillary antrum. This appearance is equivocal and could represent chronic rhinosinusitis, fungal sinusitis, or tumour. However, low signal centrally on (b) coronal STIR, surrounded by peripherally enhancing thickened mucosa on (c) coronal contrast-enhanced T1W, is consistent with a left maxillary into middle meatal mycetoma and maxillary sinusitis.

**Figure 6:**
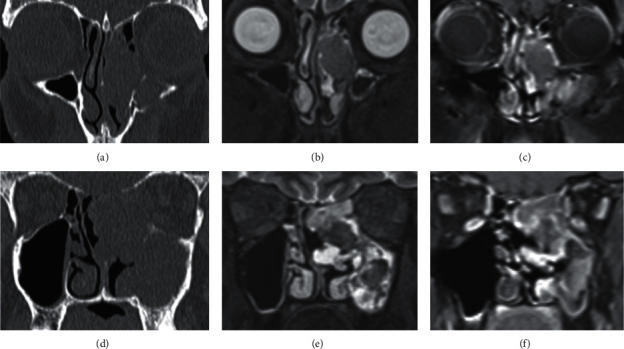
Diffuse large B-cell lymphoma in a 60-year-old male with a 4-month history of nasal blockage not responding to antibiotics. (a) Coronal unenhanced CT shows left sinonasal opacification with thinned medial maxillary sinus wall and destroyed middle turbinate. (b) Coronal STIR demonstrates a left nasal cavity lesion obstructing the maxillary sinus, which is hypointense to mucosa with heterogenous enhancement on (c) contrast-enhanced T1W. The left cribriform plate appears thinned and focally dehiscent on unenhanced CT (d); however, (e) STIR and (f) contrast-enhanced T1W show the skull base and medial orbital wall are intact.

Whereas the current standard of care imaging with CT has high sensitivity for neoplasia, justifying its use as a “screening” investigation, the high FPR hampers patient triage as the cause of UMSO is frequently not differentiated. As illustrated in Figures 5 and 6, bony remodelling on CT is an equivocal feature observed in both infective sinusitis and malignancy [16]. By contrast, MRI differentiates secretions from inflamed mucosa, benign neoplasia [17], and malignancy [18, 19]. Most neoplasms demonstrate heterogenous internal enhancement and varying levels of restricted diffusion, whereas inflamed mucosa or polyps show thin peripheral enhancement and unrestricted diffusion [14] (Figures 5 and 6).

For a significant proportion of patients (27% in this cohort) with infectious/inflammatory UMSO that could be managed medically, the improved specificity of MRI mitigates the need for diagnostic ESS. In addition, the 5/9 cases of IP correctly identified at MRI (but not CT) could have proceeded straight to definitive surgery, without the need for diagnostic ESS and biopsy. This not only reduces the risk associated with multiple episodes of anaesthesia and surgery but reduces delay to definitive treatment and relieves service demand. As the cohort of patients with UMSO resistant to medical management is usually small, the cost of additional MRI should not generally be an issue. As elective operating capacity within hospitals can be strained due to competing demands, not least due to the impact of the COVID-19 pandemic [20], interventions that reduce the requirement for multiple surgical procedures will streamline patient pathways and reduce time to treat.

**Figure 7:**
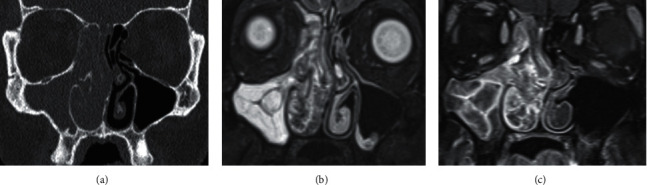
Inverted papilloma (IP) in a 32-year-old male with a 18-month history of rhinorrhoea. (a) Coronal unenhanced CT shows right unilateral maxillary sinus opacification (UMSO) with complete obstruction of the ostiomeatal unit. Bowing of the nasal septum and amorphous faint calcification could be associated with fungal disease; however, (b) coronal STIR and (c) contrast-enhanced T1W demonstrate a right nasal cavity lesion with the typical “cerebriform” appearance of IP and attachment point at the inferior orbit. Hyperintense right UMSO with mucosal enhancement indicates inspissated secretions rather than lesion extension.

In differentiating benign neoplasms requiring urgent surgery, MRI has high accuracy for diagnosing IP, where a “convoluted cerebriform pattern” is up to 100% sensitive and 87% specific [21–23] (Figure 7). The hyperostotic “bony spur” at the IP origin requires removal [24] and whilst the sensitivity for determining this origin on CT is 63% [25], MRI is both more sensitive and specific [26, 27]. This enables surgeons to plan more extensive resections, including adequate resection of the site of origin without diagnostic ESS [28], reducing reoperation [29–31].

In differentiating benign from malignant neoplasia, overlap exists at MRI in terms of enhancement pattern and diffusion characteristics [32]. Nonetheless, both require ESS for definitive treatment or biopsy confirmation, respectively [33]. Where malignancy is confirmed, the diagnostic confidence demonstrated by our results confirms the superiority of MRI over CT in delineating perineural and skull base invasion [14] or overall resection/radiotherapy margins (Figure 6).

At MRI, three of the six “false positive” cases for potential neoplasm comprised transspatial pathologies with aggressive features that overlap with neoplasia. The potentially aggressive natural history and poor prognosis of these processes justify the triage to ESS to confirm the final diagnosis (Figure 4).

No other studies have compared the diagnostic accuracy of CT with MRI for UMSO to our knowledge. Case series of UMSO have only provided qualitative descriptions of CT and MRI features in various aetiologies [16, 34–36]. Chen et al. recorded a relatively large cohort of 116 patients with UMSO but only described findings on CT. Of note, bony erosion was seen in fungal infection, benign, and malignant tumours [16]. Case series of unilateral sinonasal opacification have also presented CT findings in isolation and recommended proceeding to further investigation with biopsy [13, 37]. MRI was only utilised in a small subset of cases if neurological deficits or complications of infection were suspected [33]; thus, evaluation of its PPV for neoplastic disease was not possible.

Plain films can depict sinus opacification and air-fluid levels, but the Royal College of Radiologists has proposed that it is redundant in evaluation of sinonasal disease due to its low sensitivity and specificity [34, 38]. It is possible to diagnose rhinosinusitis on ultrasound, but the literature is not conclusive on its use in sinusitis and it plays a very limited role in characterisation of sinonasal aetiology [38]. Contrast-enhanced CT may be useful in assessment of complications of sinusitis but cannot differentiate inflammatory from malignant disease, as both can enhance [34]. Although fluorodeoxyglucose- (FDG-) PET is utilised in tumour staging and surveillance for recurrence, both tumour and inflammatory cells may demonstrate high FDG uptake, limiting its use for UMSO characterisation. Furthermore, benign tumours such as IP can have moderate FDG uptake, whilst a highly malignant tumour such as adenoid cystic carcinoma can have low FDG uptake [39]. Newer agents such as fluoro-deoxy-thymidine (FLT) have been shown to accumulate only in actively replicating cells rather than inflammatory cells, but further studies on its use in sinonasal disease are required [40].

### 4.1. Limitations

The main study limitation was small sample size. Although five patients were managed medically and hence no histological correlate was available, the final diagnosis was based on clinical findings and nasal swab as per EPOS guidelines [3]. Although the patient cohort may represent a sample in which CT and/or FNE was less informative than average (therefore necessitating MRI), resulting in a selection bias, our study demonstrates the additional diagnostic value of MRI in these uncertain cases.

Despite these limitations, the value of our data lies in the lack of studies validating the utility of MRI compared with CT in UMSO and in the high level of statistical significance of our results, indicating likely generalizable application between observers and across institutions.

## 5. Conclusions

MRI provides safe, efficient, and effective triage of patients with UMSO and by extrapolation indeterminate patterns of paranasal sinus opacification at CT. We recommend MRI evaluation in patients with complete UMSO on CT who are resistant to initial medical therapy. MRI has a higher specificity and diagnostic certainty and lower FPR for malignancy than CT, with excellent interobserver agreement. Evaluation at MRI complements CT and has the potential to reduce the need for diagnostic ESS, accelerating diagnosis and definitive management. As it informs surgical planning, it can enable better patient counselling, reduce the risk of multiple procedures, and increase cost-effectiveness of overall care. In the future, multiparametric MRI using DWI, dynamic contrast-enhanced sequences, and intravoxel incoherent motion techniques may further improve presurgical diagnostic accuracy.

## Figures and Tables

**Table 1:** 10-point diagnostic certainty criteria.

Lesion characterisation	Cystic/solid
	Polypoidal/mass
	Bone changes
	Aggressive/nonaggressive
Anatomic origin of the lesion	Maxillary ostium size
	Maxillary ostium opacification
	Maxillary origin
Involvement of secondary structures	Perineural invasion
	Soft tissue involvement
	Proximity to vessels

Each point scored as 0 (not defined) or 1 (defined).

**Table 2:** Final clinical diagnoses.

Final diagnosis	Number of cases (%)
**Infective/inflammatory (c-I)**	**29 (59.2)**
Chronic rhinosinusitis (CRS)	21 (42.9)
CRS without nasal polyps	6 (12.2)
CRS with nasal polyps (including antrochoanal polyp)	12 (24.5)
Fungal sinusitis	2 (4.1)
Mucormycosis	1 (2.0)
Mucocele	4 (8.2)
IgG4 disease	1 (2.0)
Arteriovenous malformation	1 (2.0)
Fibrous dysplasia	1 (2.0)
Pseudoepitheliomatous hyperplasia	1 (2.0)
**Neoplastic (c-N)**	**20 (40.8)**
Inverted papilloma	9 (18.4)
Cavernous haemangioma	1 (2.0)
Schwannoma	2 (4.1)
Malignant	8 (16.3)
Adenocarcinoma	1 (2.0)
Anaplastic carcinoma	1 (2.0)
Carcinoma (undifferentiated)	1 (2.0)
Lymphoma	1 (2.0)
Melanoma	1 (2.0)
Osteosarcoma	1 (2.0)
Sarcoma	1 (2.0)
Undifferentiated neuroblastoma	1 (2.0)

**Table 3:** Potentially neoplastic (N) and infective/inflammatory (I) diagnoses on CT and MRI for Observer1, Observer2, and consensus compared to final clinical diagnoses.

	Observer1				Observer2				Consensus			
	CT		MRI		CT		MRI		CT		MRI	
Final diagnosis	N	I	N	I	N	I	N	I	r-N	r-I	r-N	r-I
c-N (*n* = 20)	18	2	19	1	20	0	20	0	20	0	20	0
c-I (*n* = 29)	20	9	6	23	22	7	5	24	25	4	6	23

Chi-square statistic (*p* value) for CT versus MRI	7.5 (*p*=0.006)				13.6 (*p*=0.0002)				18.5 (*p* < 0.00002)			

**Table 4:** Diagnostic performance of CT and MRI for Observer1, Observer2, consensus, and flexible nasal endoscopy.

	Observer1		Observer2	
	CT	MRI	CT	MRI
Sensitivity	90 (68–99)	95 (75–100)	100 (83–100)	100 (83–100)
Specificity	31 (15–51)	79 (60–92)	24 (10–44)	83 (64–94)
PPV	47 (40–54)	76 (61–87)	48 (43–53)	80 (64–90)
NPV	82 (52–95)	96 (77–99)	100	100
Accuracy	55 (40–69)	86 (73–94)	55 (40–69)	90 (78–97)

	Consensus		Flexible nasal endoscopy	
	CT	MRI		

Sensitivity	100 (83–100)	100 (83–100)	85 (62–97)	
Specificity	14 (4–32)	79 (60–92)	14 (4–32)	
PPV	44 (41–48)	77 (62–87)	41 (35–46)	
NPV	100	100	57 (25–84)	
Accuracy	49 (34–64)	88 (75–95)	43 (29–58)	

Sensitivity, specificity, positive (PPV) and negative predictive values (NPV), and accuracy for potentially neoplastic diagnoses are expressed in percentages (95% confidence interval).

## Data Availability

The data used to support the findings of this study are available from the corresponding author upon request.
